# Degree of COVID-19 severity and mortality in stroke: correlation of clinical and laboratory parameters

**DOI:** 10.1186/s12868-023-00837-w

**Published:** 2024-01-13

**Authors:** Abdul Gofir, Irawan Satriotomo, Yossy Catarina Budi Nur Syamsah, Mawaddah Ar Rochmah, Tommy Rachmat Setyawan, Adika Mianoki, Raymond Aris Nimrod Alvonsius Silalahi, Dhite Bayu Nugroho

**Affiliations:** 1 Department of Neurology, Faculty of Medicine, Public Health, and Nursing, Universitas Gadjah Mada/ Dr. Sardjito General Hospital, Jalan Farmako Sekip Utara, Sleman, Mlati Yogyakarta, 55281 Indonesia; 2 Department Neurology, University of Florida, Gainesville, FL USA; 3 Department of Neurology, Dr. Soeradji Tirtonegoro General Hospital, Klaten, Central Java Indonesia; 4 Department of Internal Medicine, Faculty of Medicine, Public Health, and Nursing, Universitas Gadjah Mada, Yogyakarta, Indonesia

**Keywords:** COVID-19, Mortality, Stroke, Predictors

## Abstract

**Background:**

Stroke is one of the neurological manifestations of COVID-19, leading to a significant risk of morbidity and mortality. Clinical manifestations and laboratory parameters were investigated to determine mortality predictors in this case.

**Method:**

The case control study was conducted at Dr. Sardjito General Hospital,Yogyakarta, Indonesia, with data collected between July 2020 and August 2021. All recorded clinical and laboratory data from acute stroke patients with confirmed COVID-19 were collected. Baseline characteristics, bivariate, and multivariate analyses were assessed to determine significant predictors for mortality.

**Result:**

This study involved 72 subjects with COVID-19 and stroke. The majority experienced ischemic stroke, with hypertension as the most prevalent comorbidity. Notably, 45.8% of subjects (p < 0.05) loss of consciousness and 72.2% of exhibited motor deficits (p < 0.05). Severe degree of COVID-19 was observed in 52.8% of patients, with respiratory distress and death rates of 56.9% and 58.3%. Comparison of surviving and deceased groups highlighted significant differences in various clinical and laboratory characteristics differences. Hazard ratio (HR) analysis identified loss of consciousness (HR = 2.68; p = 0.01), motor deficit (HR = 2.34; p = 0.03), respiratory distress (HR = 81.51; p < 0.001), and monocyte count (HR:1.002; p = 0.04) as significant predictors of mortality.

**Conclusion:**

Mortality in COVID-19 patients with stroke was significantly associated with loss of consciousness, motor deficit, respiratory distress, and raised monocyte count. The risk of mortality is heightened when multiple factors coexist.

## Background

Severe Acute Respiratory Syndrome Coronavirus-2 (SARS-CoV-2) infection, also known as Coronavirus Disease 2019 (COVID-19), has become a major health problem since the end of 2019 with over 750 million confirmed cumulative cases and about 1% total deaths recorded globally [1]. Since the World Health Organization (WHO) declared that COVID-19 is no longer a public health emergency of international concern in May 2023, advancements in disease treatment and global transmission prevention, including vaccination programs, have made it possible to reach the end of COVID-19 pandemic era [2]. The WHO emphasized the decrease in COVID-19 fatalities, hospitalizations, and intensive care admissions, as well as the increase in population immunity against SARS-CoV-2 on this occasion. However, COVID-19 awareness should still be considered imperative because several surges of cases have been reported and new variants have been identified in various locations, such as the increase in COVID-19 cases in Western Pacific areas at the end of year 2022 despite the low number of cases in other parts of the world. [1]

Neurological manifestations, such as encephalitis, encephalopathy, and stroke were found in 36.4% of COVID-19 patients with involvement of central nervous system symptoms [3]. The incidence of stroke among COVID-19 patients was reported up to 3.4% in the Philippines with ischemic stroke being the most prevalent type [4]. COVID-19 increases the risk of ischemic stroke 7.8 times in the first three days after respiratory distress [5]. The pathophysiology of stroke in COVID-19 is related to viral affinity towards the Angiotensin Converting Enzyme (ACE-2) receptors, which directly causes brain neuron damage [6], activation on the immune system in the form of cytokine storms, which increases the incidence of inflammation [7], a combination of hypercoagulability and endothelial dysfunction [8], and hypoxemia due to respiratory distress. [9]

Death is more common in acute stroke patients with COVID-19 [4, 5]. In individuals with COVID-19, the risk of mortality doubles with the occurrence of acute ischemic stroke [5]. COVID-19 patients with stroke had poor outcomes due to limited admission to intensive care facilities and use of ventilators [4, 10]. Although it is uncommon, the mortality rate of hemorrhagic stroke patients with COVID-19 infection was also double compared to those without hemorrhagic stroke [11]. Mortality in stroke patients with COVID-19 is also linked to older age, dyspnea, smoking, kidney disease, hypertension, malignancy, diabetes mellitus (DM), and lung disease [4, 10]. Several laboratory markers from previous studies, such as increased D-dimer, decreased platelet count, decreased hemoglobin concentration, increased creatinine, increased Interleukin-6 (IL-6), and elevated cardiac troponin-I have become predictors of COVID-19 mortality [12]. Other predictors of COVID-19 mortality include albumin, total bilirubin, Serum Glutamic Oxaloacetic Transaminase (SGOT), Serum Glutamic Pyruvic Transaminase (SGPT), nitrogen urea (p-ES), C-reactive protein (CRP), lactate dehydrogenase (LDH) and ferritin levels. [12]

Despite various studies that have extensively investigated predictors of mortality in COVID-19 and acute stroke, separately, there is a notable gap in understanding which factors significantly contribute to the mortality risks in COVID-19 patients with stroke. Numerous studies have highlighted the role of coagulopathy, leading to a hypercoagulable state, and its adverse impact on outcomes in this specific population. For instance, elevated d-dimer levels (> 5.15 μg/ml FEU) were found to be associated with nearly three times increased mortality in hospitalized COVID-19 patients with acute ischemic stroke [13]. However, there remains a lack of exploration into other clinically and laboratory-relevant parameters that may be collinearly associated with mortality in COVID-19 patients with stroke. This study addresses this gap by examining a comprehensive set of clinical and laboratory parameters to identify mortality predictors in patients with acute stroke and COVID-19.

## Methods

### Research subject

This retrospective case–control study used medical record data at the Dr. Sardjito General Hospital in Yogyakarta, Indonesia from July 2020 to August 2021. All acute stroke patients with the evidence from non-contrast head computerized tomography (CT) scan imaging and confirmed COVID-19 from polymerase chain reaction (PCR) of SARS-Cov-2 from nasal swab sampling, age > 18 years with complete medical records covering all clinical and laboratory parameters were included in this study. Patients with missing or incomplete data were excluded.

### Research variables

The demographic information, medical history, clinical features, degree of COVID-19, laboratory results upon admission, and mortality outcome of the patients were all obtained. Data on the patient's medical history included a history of previous stroke, hypertension, diabetes mellitus, dyslipidemia, and renal insufficiency. Clinical data assessed routinely at admission included systolic and diastolic blood pressure, level of consciousness, motor deficits, aphasia, cranial nerves paresis, focal seizures, generalized seizures, any incidence of respiratory failure, and the patient's functional status with Activity Daily Living (ADL) score, Instrumental Activity of Daily Living (IADL), Barthel Index, modified Rankin Scale (mRS), and degree of neurological deficit according to the National Institutes of Health Stroke Scale (NIHSS) score, as well as the patients’ severity of COVID-19. COVID-19 severity was classified as mild, moderate, or severe based on the categorization of medical records by internal medicine specialists in accordance with the 3rd edition of the 2020 COVID-19 Management Guidelines in Indonesia [14]. Laboratory data included routine blood analysis at admission (hemoglobin, erythrocytes, leukocytes, neutrophils, lymphocytes, neutrophil-to-lymphocyte ratio (NLR), monocytes, eosinophils, basophils, and platelets), random blood glucose (RBG) level, coagulation factors: plasma prothrombin time (PPT), activated partial thromboplastin time (APTT), internationalized normalized ratio (INR), and D-dimer), markers of tissue damage (CRP, LDH); renal function biomarkers: blood urea nitrogen (BUN) and creatinine; and electrolytes (sodium, potassium, and chloride). Stroke type was determined based on the description non-contrast head computerized tomography (CT) scan.

### Statistical analysis

Statistical analysis was performed using SPSS version 23 (IBM Corp., Armonk, NY). The univariate descriptive analysis assessed the characteristics and demographics of the research subjects. The bivariate analysis compared clinical and laboratory characteristics between patients who died (deceased group) with those who survived (surviving group) at hospital release. Shapiro–Wilk analysis was used to determine the distribution of numerical data. Independent T-tests were used for data with normal distribution, whereas Mann–Whitney tests were used for non-normal distributions. Data were considered significant when *p* < 0.05. A regression analysis was performed to determine the most influential variables in the mortality of stroke patients with COVID-19. The hazard ratio was calculated by the Cox regression method with the backward system with the dependent variables being mortality and length of stay,. To demonstrate survival analysis, the significant variables were evaluated in a Kaplan–Meier model. The interval variables were then transformed into categorical variables by measuring the cut-off point with a receiver operating characteristic (ROC) curve and J Youden Index.

## Results

The demographic and clinical characteristics of all the study subjects are listed in Table 1. A total of 72 subjects were included in the study. The respondents’ mean age was 62.33 ± 12.28 years, with more males than females. The vast majority of stroke occurred for first-time occurrences. Almost every patients (90.3%) suffred an ischemic stroke. Hypertension was the most prevalent comorbidity from the history of prior diseases (63.9%). Diabetes mellitus (DM), heart disease, dyslipidemia, and renal insufficiency were all reported by < 50% of the subjects. The average blood pressure indicated systolic hypertension, whis is defined by systolic above 140 mmHg and diastolic values below 90 mmHg. The median of quantitative GCS value was 15, with a 5-point interquartile range, ranging from *compos mentis* to stuporous state. There were 33 subjects (45.8%) who experienced a loss of consciousness (GCS < 15). The majority of subjects had motor deficits (72.2%) and cranial nerve paralysis (51.4%), while only a few suffered aphasia, focal seizures, or generalized seizures. The classification of neurological deficits based on the NIHSS score revealed a moderate average of neurological deficits. More than half of the patients (52.8%) have severe COVID-19. The percentages of patients’ who experienced severe respiratory failure and death were 56.9% and 58.3%, respectively. Tables 2 and 3 show the comparison of the clinical and laboratory characteristics between the surviving and deceased groups of COVID-19 stroke patients. Essential laboratory characteristics showed increased total leukocytes, neutrophil lymphocyte ratio (NLR), coagulation factors, decreased kidney function marked by increased blood urea nitrogen (BUN) and creatinine, and liver damage indicated by increased SGOT and SGPT.

**Table 1:** Demographic, Clinical, and Laboratory Characteristic of Subjects

Characteristics	Proportion (%)	Mean ± SD/Median (IQR)
Age (years)		62.33 ± 12.28
Gender Man Woman	43 (59.7%) 29 (40.3%)	
Episode stroke New strokes Recurrent strokes	51 (70.8%) 21 (29.2%)	
Stroke type Ischemic stroke/NHS Hemorrhagic stroke/HS	65 (90.3%) 7 (9.7%)	
History of smoking	8 (11.1%)	
History of hypertension	46 (63.9%)	
History of DM	32 (44.4%)	
History of cardiac disease	16 (22.2%)	
History of Dyslipidemia	13 (18.1%)	
History of renal insufficiency	19 (26.4%)	
Systolic (mmHg)		141. 04 ± 28.63
Diastolic (mmHg)		83.51 ± 17.73
Quantitative GCS (score)		15 (10–15)
Loss of consciousness	33 (45.8%)	
Motor deficit	52 (72.2%)	
Aphasia	18 (25%)	
Cranial nerve paresis	37 (51.4%)	
Focal Seizures	2 (2.8%)	
Generalized seizures	6 (8.3%)	
Severity of COVID-19 Mild Moderate Severe	6 (8.3%) 28 (38.9%) 38 (52.8%)	
Hemoglobin (g/dl)		13.5 (11.65–15.35)
Erythrocyte (10^6^/µL)		4.61 ± 1.09
Leukocytes (10^3^/µL)		10.05 (7.39–12.74)
Neutrophils (10^3^/µL)		8.14 (5.59–10.69)
Lymphocytes (10^3^/µL)		1.06 (0.66–1.46)
NLR		7.69 (3.94–11.45)
Monocytes (10^3^/µL)		0.57 (0.29–0.85)
Eosinophils (10^3^/µL)		0 (0–0.04)
Basophils (10^3^/µL)		0.01 (0–0.11)
Platelets (10^3^/µL)		263.81 ± 122.09
PT (seconds)		15.85 (14.35–17.35)
APTT (seconds)		35.65 (29.25–42.05)
INR		1.17 (1.06–1.29)
D-Dimer (ng/mL)		1182.5 (99–3276)
CRP (mg/dl)		92.5 (35–150)
LDH (U/L)		317.5 (211–424)
Albumin (g/dL)		3.27 ± 30.50
BUN (mg/dL)		23.4 (5.88–40.93)
Creatinine (mg/dL)		1.31 (0.80–1.82)
RBG (mg/dl)		135 (42–228)
Sodium		136.5 (133–140)
Potassium		4.31 ± 0.88
Chloride		102 (98.5–105.5)
SGOT (U/L)		42.5 (21.5–63.5)
SGPT (U/L)		26.5 (12.5–40.5)
Respiratory Distress	41 (56.9%)	
Length Of Stay (LOS)		5.5 (2–9)
Mortality		
Survive	30 (41.7%)	
Dead	42 (58.3%)	

NIHSS: National Institutes of Health Stroke Scale; DM: Diabetes Mellitus NLR: Neutrophil Lymphocyte Ratio; PT: Prothrombin Time; APTT: Activated Partial Thromboplastin Time; INR: International Normalized Ratio; CRP: C-reactive protein; LDH: Lactate Dehydrogenase; BUN: Blood Urea Nitrogen; RBG: Random Blood Glucose; SGOT: Serum Glutamic Oxaloacetic Transaminase; SGPT: Serum Glutamic Pyruvic Transaminase

**Table 2:** Clinical Characteristics of the Survive vs Dead Subjects

Characteristics	Survive (n = 30)	Dead (n = 42)	p-value
Age (years)^a^	59.6 ± 13.4	64.29 ± 11.02	0.109
Gender^b^ Man Woman	17 (39.5%) 13(44.8%)	26 (60.5%) 16 (55.2%)	0.419
Episode stroke^b^ New strokes Recurrent strokes	20 (66.7%) 10 (33.3%)	31 (73.8%) 11 (26.2%)	0.432
Stroke Type^b^ Ischemic stroke/SNH Hemorrhagic stroke/SH	29 (6.7%) 1 (3.3%)	36 (85.7%) 6 (14.3%)	0.125
Systolic (mmHg)^a^	144.2 ± 25.2	138.7 ± 30.9	0.428
Diastolic (mmHg)^a^	88.6 ± 18.1	79.8 ± 16.8	0.039*
Quantitative GCS^c^ (score)	15 (13–15)	12.5 (9–15)	0.008*
Loss of consciousness^b^	8 (26.7%)	25 (59.5%)	0.006*
Motor deficit^b^	25 (83.3%)	27 (64.3%)	0.075
Aphasia^b^	7 (23.3%)	11 (26.2%)	0.783
Cranial nerve paresis^b^	15 (50%)	22 (52.4%)	0.843
Focal seizures^b^	1 (3.3%)	1 (2.4%)	0.808
Generalized seizures^b^	5 (16.7%)	1 (2.4%)	0.031*
NIHSS^c^ (score)	6 (2–10)	17 (11–23)	< 0.001*
History of smoking^b^	2 (6.7%)	6 (14.3%)	0.268
History of hypertension^b^	20 (66.7%)	26 (61.9%)	0.678
History of DM^b^	10 (33.3%)	22 (52.4%)	0.109
History of cardiac disease^b^	6 (20%)	10 (23.8%)	0.701
History of dyslipidemia^b^	9 (30%)	4 (9.5%)	0.026*
History of renal Insufficiency^b^	6 (20%)	13 (31%)	0.299
Severity of COVID-19^c^ Mild Moderate Severe	5 (16.7%) 17(56.7%) 8 (26.7%)	1 (2.4%) 11 (26.2%) 30 (71.4%)	0.001*
Respiratory distress^b^	1 (3.3%)	40 (95.2%)	< 0.001*
Length of stay (LOS)^c^	7.5 (6–9)	3 (3.5–11.5)	0.012*

^*^p value < 0.05 was considered significant, ^a^Unpaired T-test, ^b^Chi’s Square, ^c^Mann-Whitney test

NIHSS: National Institutes of Health Stroke Scale; DM: Diabetes Mellitus; NHS: Non-Hemorrhagic Stroke; HS: Hemorrhagic Stroke

**Table 3:** Laboratory Characteristics of the Survive vs Dead Subjects

Characteristics	Survive (n = 30)	Dead (n = 42)	p-value
Hemoglobin^a^ (g/dl)	13.35 (11.9–14.8)	13.85 (11.45–16.25)	0.205
Erythrocyte^b^ (10^6^/µL)	4.53 ± 0.73	4.68 ± 1.3	0.163
Leukocytes^a^ (10^3^/µL)	9.89 (6.86–12.93)	10.14 (7.62–12.66)	0.991
Neutrophils^a^ (10^3^/µL)	7.22 (4.42–10.02)	8.67 (6.07–11.27)	0.364
Lymphocytes^a^ (10^3^/µL)	1.23 (0.56–1.96)	0.99 (0.69–1.29)	0.178
NLR^a^	7.26 (2.82–11.7)	8.53 (4.69–12.37)	0.213
Monocytes^a^ (10^3^/µL)	0.69 (0.27–1.12)	0.52 (0.35–0.70)	0.027*
Eosinophils^a^ (10^3^/µL)	0.01 (0–0.1)	0 (0–0.01)	0.050
Basophils^a^ (10^3^/µL)	0.02 (0.02–0.03)	0.01 (0–0.02)	0.168
Platelets^b^ (10^3^/µL)	296.20 ± 108.15	240.68 ± 127.38	0.068
PT^a^ (seconds)	15.95 (14–17.9)	15.8 (14.5–17.1)	0.727
APTT^a^ (seconds)	39.55 (32.4–46.7)	34.85 (29.4–40.3)	0.057
INR^a^	1.16 (1–1.33)	1.2 (1.07–1.33)	0.526
D-Dimer^a^ (ng/mL)	952.5 (185–2540)	1585 (99–3689)	0.320
CRP^a^ (mg/dl)	48 (5–144)	119 (49–150)	0.028*
LDH^a^ (U/L)	311 (199.5–422.5)	342 (228.5–455.5)	0.293
Albumin^b^ (g/dL)	3.20 ± 0.67	3.32 ± 0.56	0.387
BUN^a^ (mg/dL)	18.85 (6.51–31.19)	34.25 (14.58–53.93)	0.009*
Creatinine^a^ (mg/dL)	1.19 (0.74–1.64)	1.39 (0.75–2.04)	0.105
RBG^a^ (mg/dl)	127 (66–188)	158.5 (48–290)	0.032*
Sodium^a^	137 (133.5–140.5)	136 (131.5–140.5)	0.626
Potassium^b^	4.07 ± 0.87	4.46 ± 0.87	0.065
Chloride^a^	101.5 (98.5–104.5)	102 (97.5–106.5)	0.552
SGOT^a^ (U/L)	39.5 (20–59)	44.5 (23.5–65.5)	0.203
SGPT^a^ (U/L)	25.5 (11–40)	28 (12–44)	0.740

^*^p value < 0.05 was considered significant, ^a^Mann Whitney test, ^b^Unpaired T-test

NLR: Neutrophil Lymphocyte Ratio; PT: Prothrombin Time; APTT: Activated Partial Thromboplastin Time; INR: International Normalized Ratio; CRP: C-Reactive Protein; LDH: Lactate Dehydrogenase; BUN: Blood Urea Nitrogen; RBG: Random Blood Glucose; SGOT: Serum Glutamic Oxaloacetic Transaminase; SGPT: Serum Glutamic Pyruvic Transaminase

Table 2 showed no significant differences in age, sex, or number of stroke episodes between COVID-19 stroke survivors and those who died (*p* > 0.05). Diastolic blood pressure, quantitative GCS, loss of consciousness, generalized seizures, NIHSS score, history of dyslipidemia, COVID-19 severity, respiratory distress, and length of stay were significantly different between the survive and deceased groups of patients (*p* < 0.05).

Table 3 demonstrated that deceased group had significantly lower monocyte count, higher serum CRP level, higher serum BUN level, and a higher random blood glucose level than the survivors (*p* < 0.05). All of these laboratory values were collected when patients were admitted to the hospital.

Based on the important clinical and laboratory criteria from the bivariate analysis depicted in Tables 2 and 3, the clinically valuable and significant variables (*p* < 0.25) were assessed using the hazard ratio (HR) analysis by the Cox regression method with the backward system. After cox regression analysis with backward LR system within 22 steps, there were retained significant variables which associated with the mortality risk of COVID-19 patients with stroke, as presented in Table 4.

**Table 4:** Hazard Ratio Analysis

Variable	Hazard Ratio	CI 95% (upper-lower)	p-value^a^
Loss of consciousness	2.679	1.262 – 5.687	0.010*
Motor deficit	2.336	1.089 – 5.010	0.029*
History of diabetes mellitus	0.346	0.956 – 2.752	0.022*
Respiratory distress	81.513	11.893 – 558.681	< 0.001*
Monocytes	2.502	1.064 – 5.883	0.036*
APTT	1.024	0.999 – 1.049	0.060
Creatinine	1.351	1.065 – 1.714	0.013*
Random blood glucose	1.002	1.000 – 1.005	0.032*

^*^p value < 0.05 was considered significant, ^a^cox-regression analysis with mortality and length of stay (LOS) as dependent variable, input analysis using backward system

APTT: Activated Partial Thromboplastin Time

**Fig. 1:**
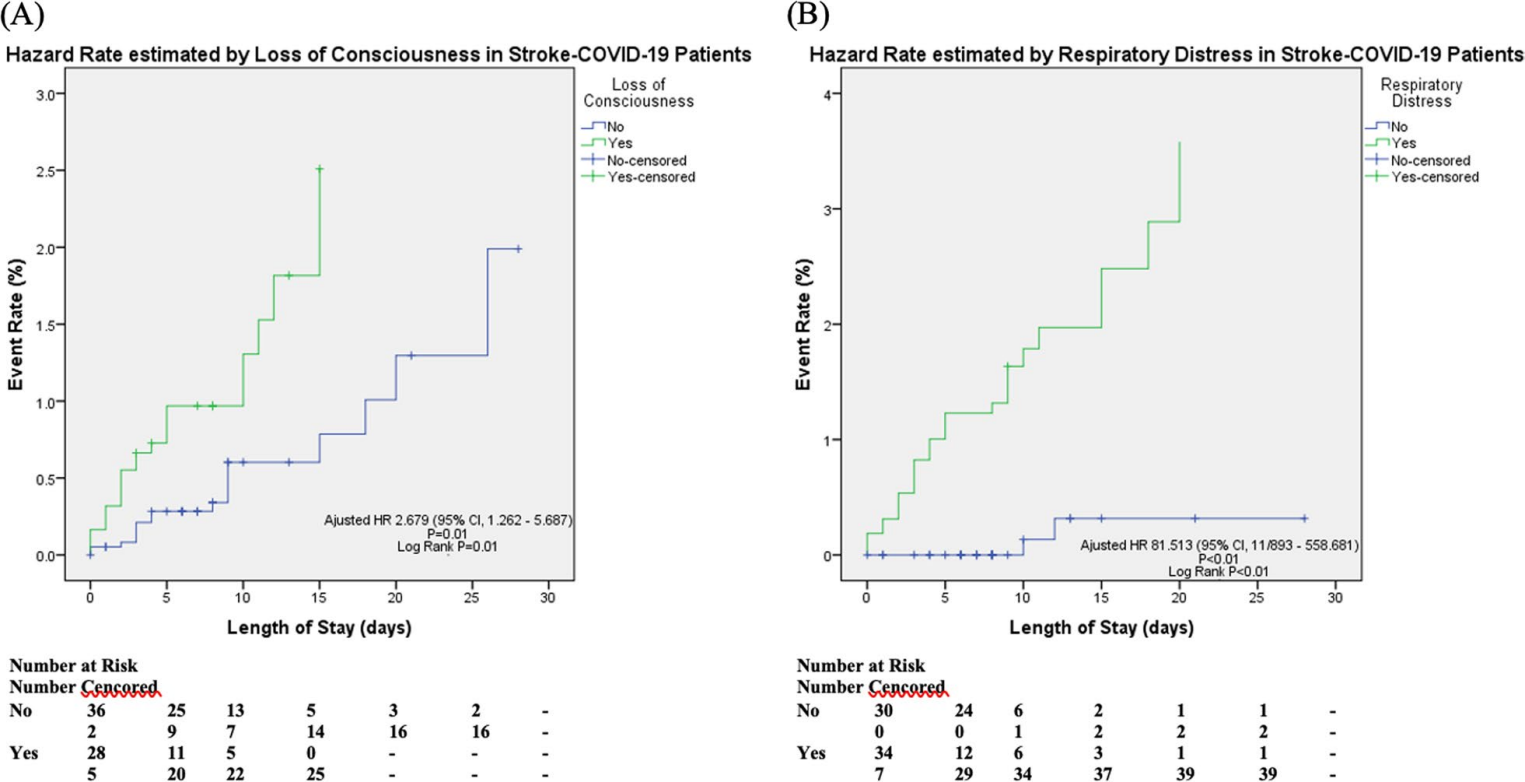
Hazard Analysis on Predictors of Mortality in Stroke Patients with COVID-19; **A** Kaplan–Meier Curve predicted by Loss of Consciousness, **B** predicted by Respiratory Distress

Except for APTT, regression analysis (Table 4) revealed that almost all of the retained significant variables were significant,. We further confirmed the important of its size effect with Area Under Curve (AUC) > 0.5, and only loss of consciousness and respiratory distress were accounted for mortality prediction. Kaplan–Meier curve (Fig. 1) was used to show the hazard rate for significant predictors in the HR model. In this study, we conducted the univariate analysis to predict the solitary risk of predictors toward mortality based on significant predictors identified in cox regression model,. We found that loss of consciousness and respiratory distress significantly increase mortality rate. Subjects who lost consciousness had a 2.68-fold greater mortality risk than those who did not. Subjects with respiratory distress had and 81.51-fold greater mortality risk than those without respiratory distress.

## Discussion

The mortality rate of patients with confirmed stroke and COVID-19 was 58.3%, according to our data (Table 1). This finding is consistent with previous studies on COVID-19 stroke patients who had a higher fatality rate than those who did not have stroke, both hemorrhagic and ischemic [4, 5, 12]. Dyspnea, smoking, cardiovascular problems, kidney and liver diseases, hypertension, diabetes, and malignancy were all associated with mortality rates in COVID-19. [15]

According to our demographic data, the population’s average age was 62.33 ± 12.28 years old. Based on data from the Centers for Disease Control and Prevention, the risk of mortality from COVID-19 rose 90 times at the age of 65–74 years and 220 times at the age of 75–84 years when compared to the age of 18–29 years. [16] Age > 65 years was also significantly associated with an increased infection fatality rate of SARS-CoV-2 infected patients in 45 countries [16]. Longer duration of comorbidities and inadequate compensation for organ function resulted in greater patient mortality [17]. Furthermore, our male subjects were more in numbers compared to female patients. According to one study, sex hormones, particularly anti-androgen and anti-estrogen, play a roles in ACE-2 protein expression in the lungs, which influence the risk of COVID-19 infection indirectly [18]. Sex disparities can also be attributed to male smoking prevalence. Nicotine has direct effects on the ACE-2 receptors, which bind to the COVID virus and cause lung tissue and brain damage. [18]

Our data revealed no link between a history of hypertension, smoking, diabetes mellitus, cardiac disease, renal insufficiency, and higher systolic blood pressure with mortality in COVID-19 patients with acute stroke. According to one recent research, uncontrolled blood pressure was not significantly associated with 30-day mortality or hospitalization incidence in COVID-19 patients with hypertension [19]. On the contrary, a history of hypertension with mean elevated blood pressure showed that systolic hypertension was associated with a significantly higher risk of mortality and an increased incidence of intensive care unit (ICU) admission in COVID-19 patients. [4, 20]

Loss of consciousness, lower GCS score, and generalized seizure were found to increase the risk of mortality in COVID-19 patients with stroke. Previous study has identified the GCS score as a risk factor for predicting mortality and allowing rapid risk stratification for SARS-CoV-2 infected stroke patients in tertiary care hospitals in low-middle-income countries where laboratory findings may be unavailable during crisis [21]. Furthermore, any neurological co-morbidities were found to have a direct significant association with stroke in patients with COVID-19, which increased mortality and ICU hospitalizations. [4]

In our study, the severity of COVID-19 and the presence of respiratory distress during hospitalization were found to have a significant relatioinship with mortality (Table 2). COVID-19 with higher severity level of may trigger cytokine cascades, exaggerated inflammatory responses, coagulation dysfunction, and organ damage, worsening the prognosis of COVID-19 stroke patients [22, 23]. Most patients with severe COVID-19 are dyspneic or hypoxemic one week after onset, with serious lung damage preventing gas exchange. [9]

As shown in Table 3, the increase in infection markers, kidney function markers, and blood glucose levels were significantly different between the surviving and deceased groups of acute stroke patients with COVID-19. Elevated CRP levels were related with higher risk of thrombosis, whereas increased LDH indicated the presence of tissue damage in COVID-19 patients [24, 25]. Neutrophils, lymphocytes, and inflammatory mediators are linked to anti-atherosclerotic vascular damage. Activated neutrophils can secrete a variety of proteolytic enzymes that stimulate endothelium and basement membrane breakdown, increasing the atherosclerotic plaque fragility and causes emboly. [26]

The laboratory parameters’ predictors with the highest ratio were a lower monocyte count and a higher BUN level at hospital admission (Table 3). Circulating pro-inflammatory stimuli in COVID-19 patients trigger the activation of blood monocytes by inducing tissue factor expression. Tissue factors expressed by activated monocytes, monocyte-derived microvesicles, and endothelial cells activate coagulation pathways, resulting in fibrin deposition and blood coagulation [27]. Several etiologies could cause high BUN level in stroke patients with COVID-19. Involvement of ACE-2 in the kidneys, which is nearly 100 times higher than in respiratory organs, deposition of viral antigen immune complexes, specific immunological effector mechanisms, and virus-induced cytokines or mediators have a direct effect on damage to kidney tissue, as well as an indirect effect via hypoxia, shock, and rhabdomyolysis. [28]

The observed coagulation blood markers showed insignificant association in predicting mortality of stroke patients with COVID-19. The population in this study consisted of 90.3% ischemic stroke patients, who developed a hypercoagulability state, as a result of thrombotic events or embolism [29]. COVID-19 infection has been linked to an increased risk for thrombosis and an increase in D-dimer levels, both of which contribute to worsening patients outcomes [30]. Whether the infection cascade causes the hypercoagulability state after the thrombotic events, or the coagulation event in stroke causes the similar state, exacerbated by COVID infection, should be further studied in a larger population in normal, stroke, COVID-19, and stroke-COVID-19 patients.

In our study, the deceased group had significantly higher hyperglycemia than the surviving group, although there was no significant difference between the groups who had previously been diagnosed with DM (Table 2 and 3). Moreover, a significant connection was discovered between the random blood glucose level and mortality in COVID-19 stroke patients, although the hazard ratio was not regarded significant (Table 4). These findings imply that hyperglycemia-induced stress may alter immunological and inflammatory responses, resulting in a worse clinical outcome in stroke patients with and without DM. [31]

After considering the discussed factors that could influence the mortality of hospitalized COVID-19 patients with acute stroke, the multivariate analysis identified four independent factors: loss of consciousness, motor deficit, respiratory distress, and monocyte count. Among these, loss of consciousness and motor deficit are clinical factors associated with neurological aspects, specifically the presence of acute stroke. While respiratory distress in COVID-19 can cause global hypoxia and loss of consciousness, its clinical manifestation is linked to central nervous system dysfunction. On the other hand, respiratory distress and monocyte count may be more closely related to the presence of COVID-19. Despite the fact that the pathogenesis of stroke is similarly link to the inflammatory response, a considerably reduced monocyte count in stroke is unusual. In summary, both the clinical and molecular manifestations of COVID-19 and stroke can result in variables that are independently associated to the mortality of COVID-19 patients with acute stroke.

Differences in the therapy administered to patients with COVID-19 during our study period resulted from evolving national treatment guidelines, which is a drawback our study. Additionally, our investigation was limited to a single health care center, focusing solely on hospitalized who were patients confirmed positive for SARS-Cov-2 through PCR at Dr. Sardjito General Hospital, a tertiary referral hospital in Yogyakarta, Indonesia. As a result, the study covered a small number of patients, with a larger proportion of severe COVID-19 cases and complex comorbidities.

In conclusion, the mortality in COVID-19 patients with acute stroke can be attributed to the clinical manifestations and laboratory parameters associated with both conditions. Several factors, independently linked to mortality in these patients, include loss of consciousness, motor deficit, respiratory distress, and lower monocyte count. The correlation to mortality is heightened when multiple factors coexist, as each might be caused by COVID-19, acute stroke, or a combination of both. Our study findings may raise clinicians' awareness of the mortality-related factors in the management of COVID-19 patients with acute stroke.

## Data Availability

The datasets used and/or analysed during the current study are available from the corresponding author on reasonable request.

## Abbreviations

COVID-19: Corona Virus Disease 2019
GCS: Glasgow Coma Scale
mRS: Modified Ranking Scale
WHO: World Health Organization
SARS-Cov-2: Severe Acute Respiratory Syndrome Coronavirus 2
ACE-2: Angiotensin Converting Enzyme
DM: Diabetes Mellitus
IL-6: Interleukin-6
SGOT: Serum Glutamic Oxaloacetic Transaminase
SGPT: Serum Glutamic Pyruvic Transaminase
p-ES: Nitrogen urea
CRP: C-reactive protein
LDH: Lactate Dehydrogenase
CT: Computerized tomography
PCR: Polymerase chain reaction
SARS-Cov-2: Severe Acute Respiratory Syndrome Coronavirus 2
ROC: Receiver operating characteristic
ADL: Activity Daily Living
IADL: Instrumental Activity of Daily Living
NIHSS: National Institutes of Health Stroke Scale
NLR: Neutrophil Lymphocyte Ratio
BUN: Blood Urea Nitrogen
PPT: Plasma Prothrombin Time
APTT: Activated Partial Thromboplastin Time
INR: International Normalized Ratio
RBG: Random Blood Glucose
IS: Ischemic Stroke
HS: Hemorrhagic Stroke
LOS: Length of Stay
HR: Hazard Ratio
